# The Influence of Perceived Social Presence on the Willingness to Communicate in Mobile Medical Consultations: Experimental Study

**DOI:** 10.2196/31797

**Published:** 2022-05-11

**Authors:** Lijuan Chen, Danyang Zhang, Mutian Hou

**Affiliations:** 1 Department of Journalism School of Humanities Shanghai University of Finance and Economics Shanghai China; 2 Department of Journalism and Communication School of Media and Communication Shanghai Jiao Tong University Shanghai China; 3 Psychological Research and Counseling Center Southwest Jiaotong University Chengdu China

**Keywords:** mobile medical consultation, mobile medical service, perceived social presence, willingness to communicate about health, COVID-19

## Abstract

**Background:**

With the rise of online health care service, there is growing discussion on the relationship between physicians and patients online, yet few researchers have paid attention to patients’ perception of social presence, especially its influence on their willingness to communicate (WTC).

**Objective:**

The goal of the research is to investigate the influence of perceived social presence (PSP) on WTC in mobile medical consultations.

**Methods:**

Participants living in Yunnan province during the period of middle to high risk of COVID-19 infection were recruited via the internet. They were assigned randomly into 2 groups interacting with a virtual physician presenting high and low levels of social presence and then asked to complete a questionnaire. Based on the theoretical framework, the study puts forward a model evaluating the relationships among participants’ PSP, communication apprehension (CA), self-perceived communication competence (SPCC), and willingness to communicate about health (WTCH) in the computer-mediated communication between virtual physicians and patients.

**Results:**

In total 206 (106 in group 1 and 100 in group 2) valid samples were gathered (from 276 log-ins) and 88.8% (183/206) of them were aged 18 to 44 years, which approximately resembles the age distribution of the main population engaging in online medical consultation in China. Independent *t* test shows that there is significant difference between the PSP of the 2 groups (*P*=.04), indicating a successful manipulation of social presence. The total effect of PSP on WTCH is 0.56 (*P*<.001), among which 74.4% is direct effect (*P*<.001). Among the indirect effects between PSP and WTCH, the mediating effect of SPCC accounts for 68.8% (*P*<.001) and the sequential mediating effect of CA→SPCC accounts for 19.2% (*P*<.001), while the mediating effect of CA alone is not significant (*P*=.08).

**Conclusions:**

This study provides a comprehensible model, demonstrating that PSP is an important antecedent of WTCH, and the sequential mediating effect of CA and SPCC found in this study also proves that in the environment of online mobile medical services, CA cannot affect communication directly. The findings will provide some practical inspiration for the popularization of online medical service, especially for the promotion of online physician-patient communication.

## Introduction

### Background

Physician-patient communication is a hot button issue in China. Web- and mobile phone–based medical consultations as a supplement to traditional outpatient services have gradually become the prevalent pathway [1,2] to consult professionals about health problems, especially during the challenge of COVID-19 [3]. Compared with the traditional way, online health care uses relatively fewer medical resources [1] and is supplementary to the “previous form of health communication...based on face-to-face interpersonal communication and linear traditional media communication” [2]. Therefore, it is worth studying the factors and mechanisms enabling patients to feel comfortable and more willing to communicate with physicians.

Willingness to communicate (WTC) is the possibility that a person chooses to communicate (especially to start a conversation) under the condition that they are free to make a choice [4]. WTC not only depends on the individual’s innate personality but the context of the communication as well [5]. Communication plays an important role in the tension between the physician and patient and the patient’s family members [6]. “Willingness to communicate about health (WTCH) represents a situational application of the WTC construct” [7]. The way the patient experiences medical care is dynamic and complex. Many factors in the process will moderate the relationship between communication and desired outcomes [8]. Physician-patient communication via the internet may have risks of uncertainty of information quality and medical advices [9]. The patient information-seeking behavior is a 1-way communication, while visiting physicians at a hospital is a kind of 2-way interaction. Patients are usually regarded as passive recipients of the health information, and they do not always actively participate in medical consultations [10,11]. However, the patient’s participation in making a medical decision is the key to patient-centered communication [8].

Many researchers have studied WTC between physicians and patients in specific situations such as organ donation [12], clinical trials [13], and sexual health [14], etc. Petrič et al [15] found that social interactions such as communication in online health communities will affect the physician-patient relationship. The willingness to accept online treatment has also been proven to partially depend on WTC online with general practitioners [16]. Published studies have proved that WTC is closely related to the context of interaction [17,18]; however, most of them only verified the correlation, and few have tried to verify the causality. In addition, for a long time communication apprehension (CA) and perceived communication competence were considered the 2 best predictors of WTC [19], but there have not been enough empirical studies to discuss the impact of this mechanism on patients’ willingness to talk about health in a computer-mediated environment.

Compared with face-to-face interactions, online physician-patient communication has particularities such as connectivity, textuality, asynchronism, and anonymity [1], which may exert a complicated effect on the online physician-patient communication process due to the lack of social cues. The theory of social presence has been widely applied in research studies of computer-mediated communication (CMC), which lacks social cues. Therefore, to fill the research gap, this study aims to explore the underlying mechanism of the influence of social presence on users’ WTC during online mobile medical services by controlled experiments. We hope to make contributions to the understanding of online patient WTC within the framework of social presence theory and shed light on practical implications for online medical platform designers to improve the consultation service.

### Theoretical Basis

Short et al [20] define social presence as the extent to which the communication medium facilitates social emotional exchange, and the extent to which a person can experience and understand another person and the underlying personal relationship. This definition is often used to depict the perception of others being there [21,22] and is often associated with concepts such as immediacy [23], intimacy [24], authenticity [25], and social copresence [26], etc. As an inherent property of the communication medium, social presence is related to the medium’s capability to convey nonverbal cues [20]. From the perspective of psychology, social presence can be described as the “warmth” of the media—namely, the capability to make people feel human warmth and sensitivity [27].

With the development of CMC, online interactions have become more immersive. Because of the relative absence of social context information and feedback, CMC hinders the transmission of cues such as personality or hints conveyed by nonverbal behaviors in face-to-face interactions [28], which reduces social presence [29]. The perception of social presence is proved to positively influence users’ trust and intention in the online environment. For instance, it can increase consumer trust in an online shopping environment, willingness to purchase online [30,31], electronic loyalty [32] and intention of continuous use [33]. Social presence is crucial for inspiring the patient’s willingness to use the service, especially in an online health community based on support exchange [34]. Peng et al [35] found that in online patient-to-physician communities, social presence and especially its characteristics that enable users to feel comfortable, safe, and warm with a sense of belonging and sensitivity will positively influence users’ information-seeking behaviors and their willingness to participate, which promotes the physician-patient relationship. In general, exploration on the effect of social presence in online health care is still inadequate compared with other fields of online medical research.

Until now, researchers had not reached an agreement on the measurement of perceived social presence. Prior studies mostly used the self-report questionnaire to measure social presence. Such measurement may show relatively higher levels of significance; however, there may be more interference factors during the process. Rourke et al [36] conducted a content analysis of online discussion. Despite the 12 resulting indicators to measure social presence, they still acknowledged the limitations of developing and testing the efficacy of an instrument to measure social presence. Social presence has been proven to be closely related to the information richness theory [37]. According to the theory, the increment of social cues will increase the capacity of potential information carrying. Hassanein and Head [38] tested and verified the forecasting effect of social presence on user attitudes toward the internet by providing texts and picture design elements with various levels of social richness on the web page. Cortese and Seo [39] manipulated the communication environment and forecasted the perceived social presence. Sia et al [40] confirmed that removing visual cues and providing anonymity reduces the perception of the level of social presence. However, relatively few existing studies have empirically investigated perceived social presence (PSP) in online health care and its effect on the mediated communication between physicians and patients.

Based on the literature review, we believe that social presence is an appropriate theoretical framework to investigate how online medical interactions with different levels of information richness influence patients’ WTC.

### Hypothesis Development and Research Model

#### Willingness to Communicate About Health

Many studies have discussed the effect of social presence on WTC online especially in the field of online learning [41,42] and revealed that students who perceive a higher level of social presence are usually more willing to communicate [43-45]. Although there is research showing that patients’ WTC may help the treatment to be more effective [46], few studies have discussed patients’ WTC in online medical services. Some scholars are interested in the relationship between social presence and WTC in the context of psychotherapy. For example, Cukor et al [47] found that in online medical services, videophones create a certain level of social presence in mental disease counseling and enable the patient to be more willing to discuss complex topics. A similar effect is expected to be found in online health care based on the theory of social presence. According to studies on the relationship between PSP and users’ willingness in other fields, we suggest the following hypothesis:

H1. Participants’ PSP is positively correlated with WTCH in online medical consultation.

#### Self-perceived Communication Competence

Communication competence is generally defined as the “cyclical process that leads to the continual refinement of one’s social communication repertoire” [48], and it is a way of dynamic understanding instead of a personality trait [49]. Since it is difficult to conceptualize or measure communication competence, most behavior-oriented studies focus on “individuals’ perceptions of competent communication behaviors” [48].

McCroskey and McCroskey [50] define self-perceived communication competence (SPCC) as an individual’s evaluation of their confidence in their ability to communicate. It is the individual’s self-perception instead of their actual competence or skill that initiates WTC [51]. The effect of communication competence has been verified in the medical field [52,53]. The Medical Communication Competence Scales developed by Cegala et al [54] help to further study the physician-patient interaction in medical interviews. In the CMC environment, patients’ competence in medical communication will help them describe the physical condition to the physician [55].

Communication competence is seldom examined with social presence. Wrench and Punyanunt-Carter [56] demonstrated that there is a positive correlation between CMC competence and CMC presence. Although CMC competence is not equivalent to communication competence, it has been proven that CMC competence embodies the notion of communication competence. In addition, SPCC has been proven to have significant influence on people’s WTC [57], and an individual’s perception of their competence can overcome the influence of their actual competence on the WTC [58]. When an individual is motivated to communicate competently, their perception of competence increases [59] and their WTC will grow accordingly.

Therefore, we hypothesize the following:

H2. Participants’ SPCC mediates the relationship between PSP and WTCH in online medical consultation.

#### Communication Apprehension

Studies on CA started from the 1970s. It refers to “an individual’s level of fear or anxiety associated with either real or anticipated communication with another person or persons” [60]. In general, CA is a large topic and has various definitions. It is also conceptualized as (1) the personality trait an individual has in communication, which is enduring in communicative situations, or (2) the state of experience, which is situation-specific [61,62]. There are fewer people with trait CA than people with state CA [63].

Studies have proven the mutual influence between CA and social presence [39]. Ayres and Hopf [64] found that the perception of a human being can help to reduce CA. On the other hand, people who have experienced a high level of apprehension tend to perceive a lower level of social presence [56,65].

Few studies connect the application of CMC with CA, yet Burke et al [66] found that the application of CMC is strongly connected with the computer-mediated apprehension. People with higher level of CA perceive a lower level of social presence [39] and are more inclined to engage in CMC discussions [66]. In telemedicine, the presence of credible medical service providers or family members will help to reduce patients’ apprehension effectively [67]. In addition, CA has significant influence on individuals’ behaviors and motivations [68], especially on their WTC [39,66,69], considering both the amount and quality of the communication [70]. People with high levels of CA will try to keep silent or talk as little as possible to avoid communication, while those with low levels of CA usually seek opportunities to communicate [71]. It does not necessarily mean that people with high levels of CA will totally avoid CMC, yet compared with those with low levels of CA, their WTC online does not seem to be very strong. Thus, we hypothesize the following:

H3. Participants’ CA mediates the relationship between PSP and WTCH in online medical consultation.

A high level of CA will lead to more negative evaluation of self-competence [72]. McCroskey et al [73] found that CA is negatively associated with SPCC, which has been further verified in other empirical studies [74,75]. People who are more apprehensive of communication tend to believe that they are less competent communicators [76,77]. Based on the findings of the above research studies, we hypothesize the following:

H4. Participants’ CA and SPCC sequentially mediate the relationship between PSP and WTCH in online medical consultation.

**Figure 1:**
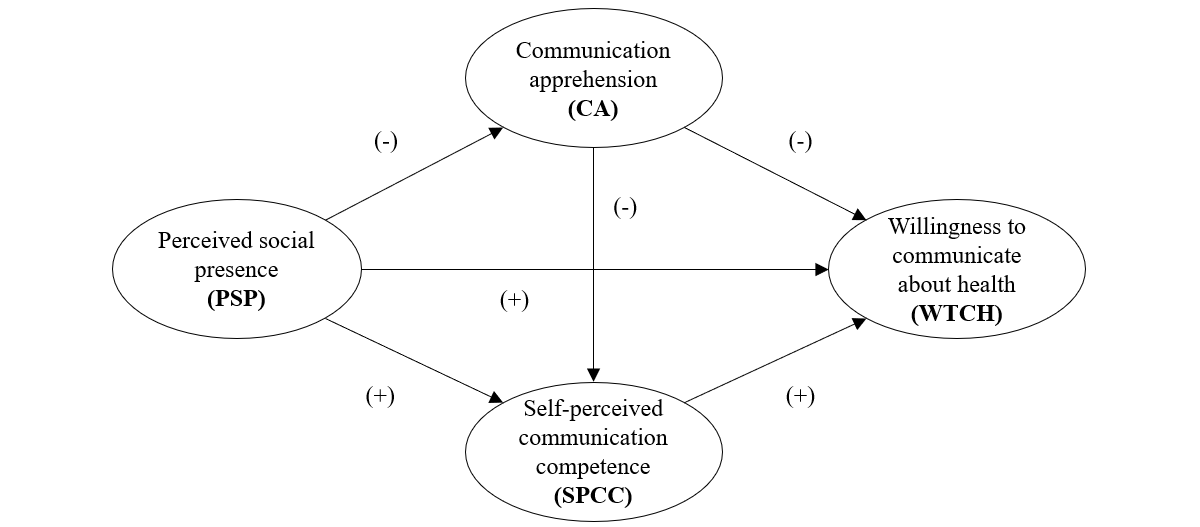
Research model.

Figure 1 presents the proposed research model which depicts both the direct relationship and the sequential indirect relationship between PSP and WTCH through CA and SPCC.

## Methods

### Participants

We cooperated with a legally registered data company that helped us contact and recruit qualified participants living in Yunnan province via the internet with compensation. Only participants with their mobile IP located in Yunnan could take part in the experiment and complete the questionnaire. In total, 299 people participated in the experiment with 276 completing the whole process. However, 70 participants failed to pass the reverse coding test. Therefore, 206 valid *samples were finally collected (each compensated with 4 renminbi [RMB]), yielding a valid participation rate of 68.9% (see Table 1 for participant demographic information). According to a report by China Industrial Information [78], users aged 18 to 44 years account for the majority of all users on online medical platforms, with male users being 54.6% and female users being 45.4%. In our experiment, participants aged 18 to 44 years account for 88.8% (183/206), and male users and female users are 59.2% (122/206) and 40.8% (84/206), respectively, which is basically in line with the users on online medical platforms in China.

**Table 1:** Participant demographics.

Characteristics			Value, n (%)
**Gender**			
	Female	84 (40.8)	
	Male	122 (59.2)	
**Age (years)**			
	<18	11 (5.3)	
	18-24	79 (38.4)	
	25-34	69 (33.5)	
	35-44	35 (17.0)	
	>44	12 (5.8)	
**Education level**			
	Primary school	1 (0.5)	
	Junior high school	25 (12.1)	
	High school	38 (18.5)	
	Junior college	58 (28.2)	
	Bachelor	75 (36.4)	
	Master	9 (4.4)	
	Doctor	0 (0)	
**Monthly income, RMB^a^ (US$), n (%)**			
	<1000 (154)	15 (7.3)	
	1000-2000 (154-308)	7 (3.4)	
	2000-5000 (308-772)	61 (29.6)	
	5000-10,000 (772-1544)	102 (49.5)	
	10,000-100,000 (1544-15,440)	20 (9.7)	
	>100,000 (>15,440)	1 (0.5)	

^a^RMB: renminbi.

### Ethics Approval

The experiment was approved (H2021099I) by the Institutional Review Board for Human Research Protections of the Shanghai Jiao Tong University on March 23, 2021.

### Materials

**Figure 2:**
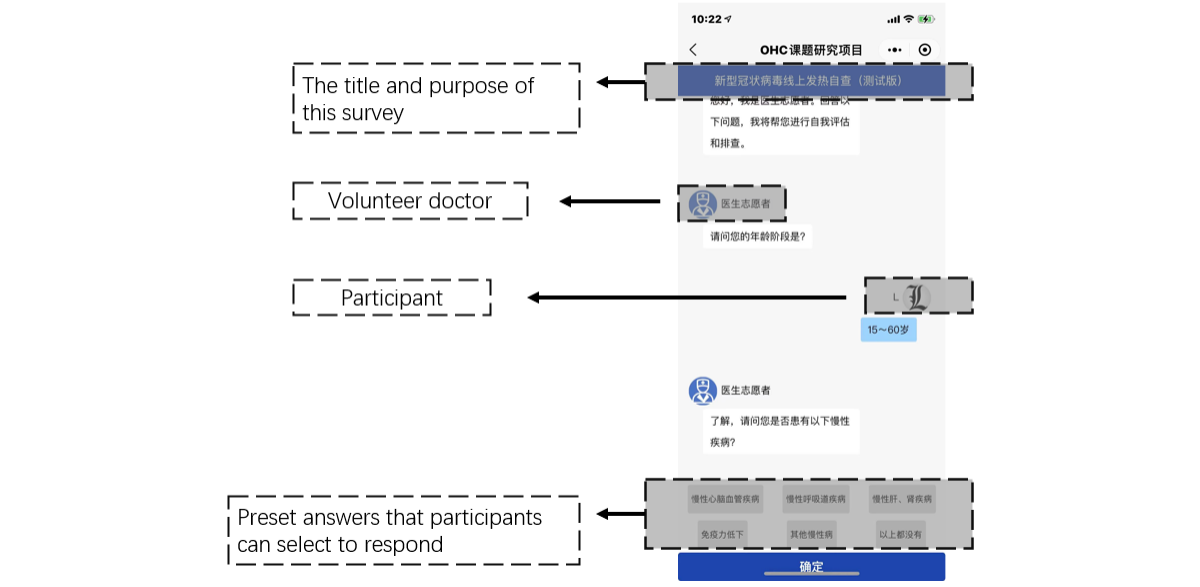
Interface for group 1 with a low level of social presence.

**Figure 3:**
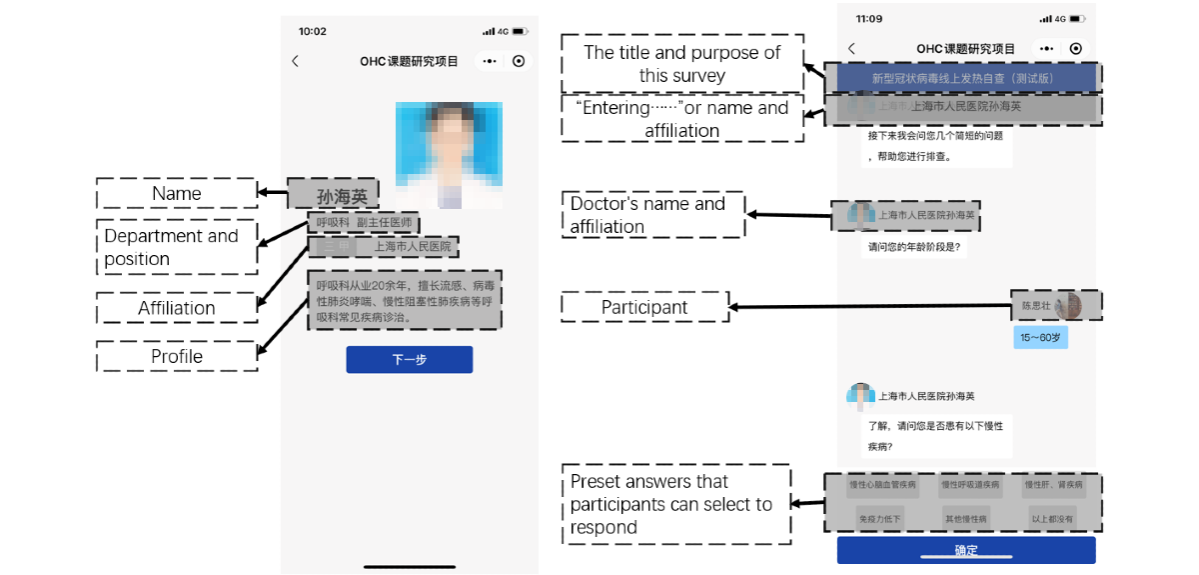
Interface for group 2 with a high level of social presence.

Our experiment used the stimuli investigated in our pretest. Group 1 was presented with a physician using a default profile photo in the system without any personal or professional information (Figure 2) while participants in group 2 were presented with a page illustrating the physician’s profile including a photo, name, position, institution, expertise, etc, before the experiment started. Interactive experiences such as some random waiting time, a sign on the top indicating that the physician is entering the name and profile photo presented in the dialog box, etc, were also added in group 2 (Figure 3). Except for the above differences, the experiment was conducted under the same system settings for the 2 groups. In order to keep the consistency and effectiveness of the independent variables, the responses and diagnosis from the physician in the dialogs were based on the patient’s selection of preset options.

We conducted a trial test on the stimuli of the experiment on November 11, 2020. We randomly invited 35 students (aged 17-23 years; male 57% [20/35], female 43% [15/35]) in a cafeteria on the campus of a major university in China to participate in our online experiment. The result of the independent samples *t* test shows that the levels of PSP are significantly different under 2 groups of stimuli (group 1: 2.68 [SD 0.39], group 2: 3.38 [SD 0.52]; *P*<.001).

### Design and Procedure

We have designed a 1-factorial experiment on 2 groups with the manipulation of social presence level. Participants were assigned randomly into 2 groups: group 1 with a low level of social presence and group 2 with a high level of social presence. Beside the stimuli, other system settings were the same for the 2 groups. Before the experiment started, participants were asked to provide demographic information and then, to improve the authenticity of the task, participants were invited to a pretest of an online system to self-check the potential risk of COVID-19 infection. Participants were informed that the test controller was expecting their honest feedback to further optimize the system, but the result of the test could not be regarded as clinical evidence. In the experiment, there was a reminder for the participants stating “If you feel sick, you need to visit a physician as soon as possible.” On March 30, 2021, 6 new cases of COVID-19 were confirmed in Yunnan province, the first since September 2020 when the number of local confirmed cases fell to zero. Our experiment (H2021099I) ran from April 8-21, 2021, because some areas in Yunnan were rated as medium to high risk areas throughout the period, suitable for the experiment since people living in Yunnan might be concerned about the risk of infection. To simulate the mobile medical service environment, our experiment was conducted entirely on smartphones involving 2 groups of participants (106 in group 1 and 100 in group 2). The experiment was followed by a questionnaire.

### Measurements

#### Social Presence

We adopted the 5-item PSP scale [30], which is frequently applied to examine the PSP in CMC. The scale ranges from 1 (strongly disagree) to 5 (strongly agree).

#### Communication Apprehension

We developed the 5-item Patients’ Report of Communication Apprehension with Physicians proposed by Ayres et al [79] based on personal perceived communication apprehension. The scale measures patients’ CA in physician-patient interactions on a scale from 1 (strongly disagree) to 5 (strongly agree).

#### Self-perceived Communication Competence

We developed the Patients’ Self-competence Items Comprising the Medical Communication Competence Scale proposed by Cegala et al [54], ranging from 1 (strongly disagree) to 5 (strongly agree).

#### Willingness to Communicate About Health

We developed the WTCH scales proposed by Wright et al [80]. This scale has 10 items divided into 2 dimensions, provider and nonprovider. It is applied to measure whether a patient is willing to communicate about their health status with different people. Since the experiment was conducted entirely on smartphones, we used the 5 items from the provider dimension only, ranging from 1 (strongly disagree) to 5 (strongly agree).

#### Willingness to Communicate

We tried to control the effects of personality types and developed the WTC scales proposed by McCroskey [81]. In this scale, WTC includes 4 types of communicative situations and 3 kinds of audiences. Based on our experiment, we adopt the 4 items associated with the investigation of the subscores related to strangers. Participants indicated the probability they would choose to communicate by reporting the percentage from 0 (never) to 100 (always).

### Validity and Reliability Check

The 4 constructs (PSP, CA, SPCC, and WTCH) in the questionnaire were all developed from scales in prior studies, and the content validity of these constructs has been repeatedly verified (see Multimedia Appendix 1). In addition, in our pretest we invited 35 participants on campus to give us some advice on the understanding, wording, and readability of the items after they completed the experiment and the questionnaire. Their suggestions were fully considered and helped us to reach the final version of the questionnaire (Multimedia Appendix 2).

Confirmatory factor analysis was run in SPSS AMOS (version 23.0, IBM Corp) to check the convergent validity and reliability of the construct. Confirmatory factor analysis fit information indicated that the measurement model was acceptable (*χ*^2^/df=1.93, root mean square error of approximation=0.07, comparative fit index=0.92, Tucker-Lewis index=0.90, incremental fit index=0.92; Table 2). According to Fornell and Larcker [82], when the average variance extracted (AVE) of the construct is equal or greater than 0.50 and its construct reliability (CR) is greater than 0.70, the construct passes the convergent validity test. The standard load factors of the items in construct PSP ranged from 0.72 to 0.82 (*P*<.001, AVE=0.57, CR=0.87). The factor of CA ranged from 0.58 to 0.84 (*P*<.001, AVE=0.52, CR=0.81). Since the 6 items in construct SPCC are comprised of 2 dimensions (items 1 to 4 from 1 dimension and items 5 and 6 from the other), they were analyzed in 2 separate factors. The standard load factors of items 1 to 4 ranged from 0.70 to 0.94 (*P*<.001, AVE=0.62, CR=0.76), while the factors of items 5 and 6 ranged from 0.76 to 0.81 (*P*<.001, AVE=0.62, CR=0.89). The standard load factor of the items in construct WTCH ranged from 0.66 to 0.80 (*P*<.001, AVE=0.52, CR=0.84). The standard load factor of the items in construct WTC ranged from 0.54 to 0.87 (*P*<.001, AVE=0.51, CR=0.80). Therefore, the convergent validity and CR of our questionnaire meets the requirement.

**Table 2:** Results of confirmatory factor analysis.

Factor and item			Std^a^ loading		Convergent validity		
					CR^b^		AVE^c^
**PSP^d^**			—^e^		0.87		0.57
	PSP1	0.74		—		—	
	PSP2	0.72		—		—	
	PSP3	0.76		—		—	
	PSP4	0.82		—		—	
	PSP5	0.74		—		—	
**CA^f^**			—		0.81		0.52
	CA1	0.79		—		—	
	CA2	0.84		—		—	
	CA3	0.58		—		—	
	CA4	0.63		—		—	
**SPCC^g^ (1-4)**			—		0.89		0.68
	SPCC1	0.86		—		—	
	SPCC2	0.94		—		—	
	SPCC3	0.70		—		—	
	SPCC4	0.77		—		—	
**SPCC (5-6)^h^**			—		0.76		0.62
	SPCC5	0.81		—		—	
	SPCC6	0.76		—		—	
**WTCH^i^**			—		0.84		0.52
	WTCH1	0.69		—		—	
	WTCH2	0.69		—		—	
	WTCH3	0.80		—		—	
	WTCH4	0.66		—		—	
	WTCH5	0.74		—		—	
**WTC^j^**			—		0.80		0.51
	WTC1	0.81		—		—	
	WTC2	0.58		—		—	
	WTC3	0.54		—		—	
	WTC4	0.87		—		—	

^a^Std: standardized.

^b^CR: construct reliability.

^c^AVE: average variance extracted.

^d^PSP: perceived social presence.

^e^Not applicable.

^f^CA: communication apprehension.

^g^SPCC: self-perceived communication competence dimension 1.

^h^SPCC (5-6): self-perceived communication competence dimension 2.

^i^WTCH: willingness to communicate about health.

^j^WTC: willingness to communicate.

## Results

### Manipulation Check

The interfaces of our online experiment were designed to present 2 levels (high and low) of social presence. The independent samples *t* test was applied to examine whether the manipulation successfully triggered different levels of social presence as perceived by participants. The test proved that the PSP of group 2 was significantly higher than that of group 1 (*P*=.04, *F*=0.01, *t*=–2.12). Table 3 shows the results of the *t* test.

**Table 3:** Results of independent samples *t* test.

Group	Social presence level	Mean (SD)	*F* score	*t* score	*P* value
1	Low	3.85 (0.77)	0.01	–2.12	.04
2	High	4.07 (0.75)	—^a^	—	—

^a^Not applicable.

### Hypothesis Testing

Based on the proposed model of this study, model 6 described in the bootstrap methods of SPSS PROCESS developed by Hayes [83] was applied to analyze the sequential mediating effect of the model. In this study, the average score of the items in the construct was calculated to represent the value of each construct. In the model, PSP is the independent variable (X), WTCH is the dependent variable (Y), CA is mediator 1 (M_1_), and SPCC is mediator 2 (M_2_).

#### Correlation Test

Based on the proposed model of this study, a partial correlation test controlling the effects of age, gender, education level, income level, and WTC was conducted to examine the intercorrelations among the 4 variables. Results in Table 4 show that the intercorrelations among all the variables are significant (*P*<.001) and the signs of the coefficients are consistent with the model prediction.

The total, direct, and indirect effects between PSP and WTCH with the mediation of CA and SPCC are calculated by model 6, controlling the effects of age, gender, education level, income level, and WTC.

**Table 4:** Partial correlation analysis controlling age, gender, education level, income level, and willingness to communicate.

	PSP^a^	CA^b^	SPCC^c^	WTCH^d^
PSP	—^e^	—	—	—
CA	–.23	—	—	—
SPCC	0.37	–.40	—	—
WTCH	0.64	–.35	0.6	—

^a^PSP: perceived social presence.

^b^CA: communication apprehension.

^c^SPCC: self-perceived communication competence.

^d^WTCH: willingness to communicate about health.

^e^Not applicable.

#### Total Effect

The regression effect between PSP (X) and WTCH (Y) is significant. PSP positively affects WTCH, supporting H1. The total effect of PSP on WTCH is 0.56 (*P*<.001, *t*=11.63, *R*^2^=0.42, *F*=23.71).

#### Direct Effect

According to the results calculated based on model 6, the direct effect of PSP on WTCH accounts for 74.37% (*P*<.001, effect=0.41, *t*=9.30) of the total effect, indicating that 25.63% of the total effect is indirect.

#### Indirect Effect

The results in Tables 5 and 6 show that the coefficient of PSP→CA, PSP→SPCC, CA→SPCC, PSP→WTCH, and SPCC→WTCH are all significant while the coefficient of CA→WTCH is not significant, indicating that the indirect pathway of PSP→SPCC→WTCH is supported (supporting H2) and the sequential mediation of the pathway of PSP→CA→SPCC→WTCH is supported (supporting H4), but the indirect pathway of PSP→CA→WTCH is not supported (rejecting H3). It indicates that CA alone cannot mediate the relationship between PSP and WTCH. Instead, CA affects WTCH only through the mediating effect of SPCC.

Table 7 summarizes the indirect effects of the 3 pathways. According to Hayes, the pathway is significant when the BootLLCI and BootULCI are both above zero or below zero [83]. Therefore, the pathway of PSP→CA→WTCH is not significant while the other 2 are significant, repeating the results from the equations. The effect of the pathway of PSP→SPCC→WTCH accounts for 68.75% of the total indirect effect and for the pathways of PSP→CA→SPCC→WTCH and PSP→CA→WTCH, the ratios are 19.24% and 12.01%, respectively. It can be inferred that the effect through the mediator SPCC accounts for 87.99% of the total indirect effect, indicating that SPCC plays a more important role in this model.

**Figure 4:**
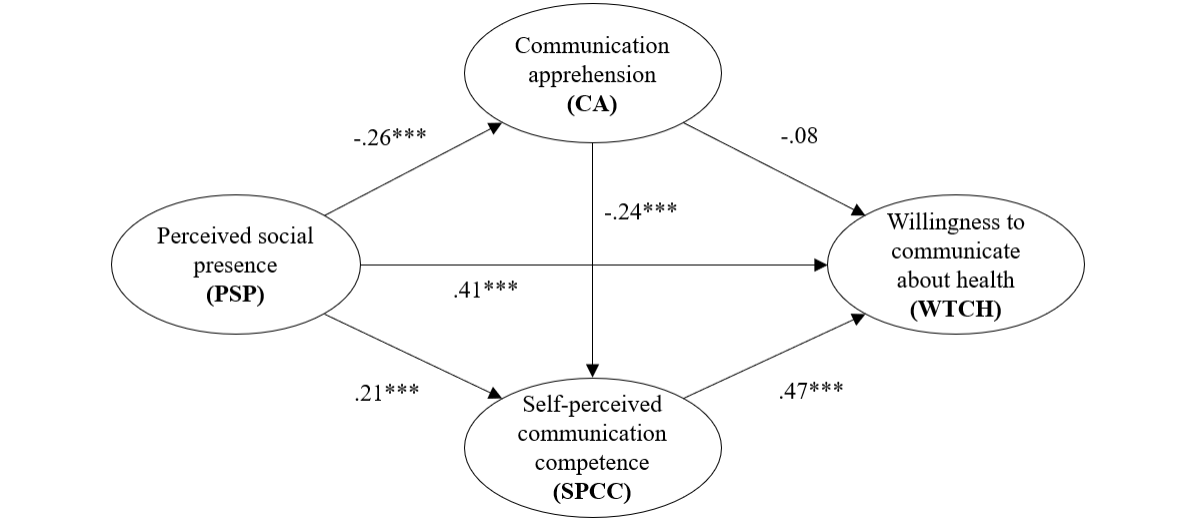
Final mediation model. ***P<.001.

Based on the coefficients of the causal paths illustrated in Tables 5 and 6, the final model is presented in Figure 4.

**Table 5:** Summary of the coefficients and the models controlling age, gender, education level, income level, and willingness to communicate.

	CA (M_1_)^a^				SPCC (M_2_)^b^				WTCH (Y)^c^		
	Coeff.	*t*	*P* value	Coeff.		*t*	*P* value	Coeff.		*t*	*P* value
PSP (X)^d^	–.26	–3.38	<.001	0.21		4.54	<.001	0.41		9.3	<.001
CA (M_1_)	—	—	—	–.24		–5.14	<.001	–.08		–1.75	.082
SPCC (M_2_)	—	—	—	—		—	—	0.47		7.2	<.001

^a^CA (M_1_): communication apprehension as mediator 1.

^b^SPCC (M_2_): self-perceived communication competence as mediator 2.

^c^WTCH (Y): willingness to communicate about health as Y.

^d^PSP (X): perceived social presence as X.

**Table 6:** Model summary of communication apprehension, self-perceived communication competence, and willingness to communicate about health as outcome variables.

	CA (M_1_)^a^	SPCC (M_2_)^b^	WTCH (Y)^c^
*R^2^*	0.11	0.29	0.57
*F* score	3.92	11.48	33.05
*P* value	.001	<.001	<.001

^a^CA (M_1_): communication apprehension as mediator 1.

^b^SPCC (M_2_): self-perceived communication competence as mediator 2.

^c^WTCH (Y): willingness to communicate about health as Y.

**Table 7:** Summary of the mediation pathways.

Mediation pathways	Effect	BootLLCI^a^	BootULCI^b^
Total	0.14	0.087	0.208
PSP→CA→WTCH	0.02	–0.001	0.048
PSP→SPCC→WTCH	0.10	0.054	0.150
PSP→CA→SPCC→WTCH	0.03	0.009	0.052

^a^BootLLCI: bootstrap lower limit confidence interval.

^b^BootULCI: bootstrap upper limit confidence interval.

## Discussion

### Principal Findings

Prior research models consider that PSP is influenced by CA in computer-mediated environments; to be specific, people with a higher level of CA will experience a lower level of PSP [39,56]. However, this is the opposite of the causality shown by our experiment results. Our research finds that the level of PSP will negatively influence people’s CA, yet the causality of this influence has been proven by Burke et al [66] to be the opposite—people experiencing a high level of CA tend to be involved in CMC discussions with a low level of social presence. In addition, Burke et al [66] found that PSP is negatively associated with WTCH, which is also the opposite of our results that clearly show a positive relationship between PSP and people’s communication willingness mediated by computers.

Prior literature has demonstrated the correlation between SPCC and WTCH [84-86], and such correlation is also applicable in the context of online medical communication studied in this research. Further findings show that SPCC mediates the relationship between PSP and WTCH. As an individual perception, SPCC is a key factor affecting users’ WTC in the CMC environment. It seems that SPCC is less associated with the individuals’ innate and actual communication skills but is more dependent on their perception of their abilities. And such perception will directly influence users’ WTC by increasing or decreasing social cues in the communication environment.

Beside the mediating effects of CA and SPCC discussed in this study, we found that the effect of CA on WTCH is exerted entirely through the mediating effect of SPCC. The perspective that health communication competence is one of the best predictors of patients’ WTC [87] has been proven again. Competence is a sociopsychological concept. It is more like a response to a specific situation or social context to achieve the communicative goals [88]. McCroskey [51] believes that whether people decide to start a conversation usually depends on self-perceived rather than actual communicative competence. People experiencing a high level of CA will decrease their evaluation of their communicative competence [72]. The sequential mediating effect of CA and SPCC found in this study also proves that in the environment of online mobile medical services, CA cannot hinder or facilitate communication directly—instead, it should transfer its effect to SPCC. If participants experience a higher level of CA in the experiment, their SPCC will be decreased accordingly, thus showing a lower WTC.

### Comparison With Prior Work

Although prior studies have examined and confirmed the influence of social presence in the medical field [34,35], due to the lack of an accepted definition and difficulty of measurement, few empirical studies have explored the relationship between social presence and WTC, let alone the exploration of the causal relationship between them. Thus, our study has supplemented current research results to a certain extent by conducting the experiment on a more diverse group of people.

In prior studies, the interrelationships among CA, SPCC, and WTC have received much attention. However, the difference in the communication performance of people with different levels of CA, especially the difference in WTC under the influence of online and offline CA and the different levels of CA when facing different people, have not been fully discussed. Some studies hold that face-to-face CA seems to motivate people to communicate online [89]. In the CMC environment which is lower in media richness, what is the influence of the state CA on WTC? In addition, the offline SPCC is decisive for the individual’s WTC. Will the online SPCC in some specific situations vary with the increase or decrease in social cues? To answer these questions, we simulates the online mobile medical environment and manipulates participants’ PSP by stimulating their imagination of interaction and providing actual interactions with the others, which supplements existing studies of the antecedents of WTC in the computer-mediated environment. Although our results show that the mediating effect of CA on the relationship between PSP and WTCH is not supported, there are some valuable findings in our research.

### Practical Implications

Our study will provide some practical inspiration for the popularization of online medical service in the future, especially for the promotion of online physician-patient communication. To construct a friendly environment for medical communication, the program designer should fully take users’ PSP into consideration. For online patients, if they have worries or lack confidence in the CMC with physicians, their SPCC will be influenced and their WTCH conditions will be affected accordingly. Given the important role of SPCC, online medical service providers should not only help users to acquire substantial communication skills, but also build a friendly consultation environment. Interactive elements enabling users to feel more confident can be applied to optimize the interface (eg, a typing prompt function to suggest expressions users may proceed with in the conversation). When users feel more confident about their competence to initiate and continue an effective communication, they are more likely to be engaged in online consultation. To take care of users’ CA, which is an influencing factor of SPCC, online health service providers should try to ease users’ stress and enhance their sense of security by scientific communication skills catering for users’ emotions. Experiencing a lower level of CA will result in an increase in SPCC, which will encourage users to engage in online health communication.

### Limitations

First, concerning our research model, the experiment only investigates the users’ WTC, and the amount of actual communication (or expressions) has not been measured. In fact, despite their unwillingness, people with a high level of CA may not have poor performance in communication [70]. Second, our experiment only investigates the text communication between physicians and patients online. Users’ WTC online through audio or video is also worth studying.

Concerning the design of the experimental stimuli, both the self-examination of the risk of COVID-19 infection and the time of conducting the experiment are cross-sectional and have their inherent limitations. If the participant wants to have medical services online and interact with a real physician, their communication will adjust to the feedbacks from each other. However, in order to control the manipulation of PSP, we have eliminated the personalized feedback from the simulated online physician. Our experiment is based on the Chinese cultural context and medical conditions. If the experiment is conducted with a more international and diverse group of participants, can the findings of this study be repeated? More proof is needed.

Finally, our study focuses more on patients; however, it has been proven that the physician-patient communication is more physician-centric in online medical services than in face-to-face consultations [46]. If researchers are interested in the field of online physician-patient communication, especially the patient’s WTC, we suggest they explore more on physician-centric studies.

### Conclusions

The computer-mediated environment was once considered as an inactive medium for the communication of health problems. Responding to the theory of social presence, we have verified the pathway of social presence’s influence on patients’ WTC. Specifically, this study examined the indirect effects of the 2 parallel mediators. The results of the experiment show that although only the indirect path via SPCC is supported, some innovative findings can still be drawn. Considering the diverse definitions of social presence, a convincing measurement remains to be developed and tested. Our experiment has proved that the increment of social cues will positively influence the degree of social presence perceived by participants in mobile medical consultations. When provided with more personal information on the physician and more interactivity, participants will perceive the social presence to be more prominent. Otherwise, there will be subtle perception of social presence. Given the identification of a reliable manipulation that can be applied to measure social presence as the embodiment of the authenticity of others in communication, future research studies can focus more on evaluating the antecedents and consequences of social presence. For direct effect, the results demonstrate that patients’ WTCH is directly influenced by PSP. For indirect effect, patients’ WTC will increase by the higher level of their SPCC. However, a lower level of CA will not lead to an increased level of WTC, which is inconsistent with prior studies and contributes to the literature of social presence theory in the field of health communication. In general, this study developed and explored the concept and analysis framework of PSP that affects patients’ WTC with physicians about their health conditions in the context of mobile health care and verified the mediating effect of online experiential interaction between physicians and patients on the latter’s WTC. The research on the impact of PSP on patients’ willingness to talk about health online is only a preliminary attempt. The process and outcome of online physician-patient communication are affected by multiple factors that need to be explored more fully in the future.

## Appendix

Multimedia Appendix 1 Construct items and sources.

Multimedia Appendix 2 Questionnaire.

## Abbreviations

AVE: average variance extracted
CA: communication apprehension
CMC: computer-mediated communication
CR: construct reliability
PSP: perceived social presence
RMB: renminbi
SPCC: self-perceived communication competence
WTC: willingness to communicate
WTCH: willingness to communicate about health
