# Supplementary material for: The Influence of Perceived Social Presence on the Willingness to Communicate in Mobile Medical Consultations: Experimental Study
Source: J Med Internet Res. 2022 May 11;24(5):e31797. doi: 10.2196/31797 (PMC9133978; doi:10.2196/31797)
Supplement: Multimedia Appendix 1 [file jmir_v24i5e31797_app1.doc]

## Multimedia Appendix 1

Construct items and sources.

| Item | Wording | Source |
| --- | --- | --- |
| **Perceived social presence (PSP)** | |  |
| PSP_1 | There is a sense of human contact when communicated with the online physician. | Gefen and Straub, 2003 |
| PSP_2 | There is a sense of personalness from the online physician. |
| PSP_3 | There is a sense of sociability when communicated with the online physician. |
| PSP_4 | There is a sense of human warmth when communicated with the online physician. |
| PSP_5 | There is a sense of human sensitivity when communicated with the online physician. |
| **Communication apprehension (CA)** | |  |
| CA_1 | While participating in a conversation with a new online physician I am not nervous. | Ayres et al., 1996 |
| CA_2 | I was very tense and nervous when communicating with this online physician. |
| CA_3 | I am not afraid to speak up in medical interviews with this online physician. |
| CA_4 | Overall, I am very calm and relaxed when talking to this online physician. |
| **Self-perceived communication competence (SPCC)** | |  |
| SPCC_1 | I did a good job of presenting important history associated with my medical problem. | Cegala et al., 1998 |
| SPCC_2 | I did a good job of describing the symptoms of my medical problem. |
| SPCC_3 | I did a good job of answering the doctor’s questions thoroughly. |
| SPCC_4 | I did a good job of answering the doctor’s questions honestly. |
| SPCC_5 | I did a good job of contributing to a trusting relationship |
| SPCC_6 | I did a good job of being open and honest |
| **Willingness to communicate about health (WTCH)** | |  |
| WTCH_1 | Comfortable talking about health with healthcare providers. | Wright et al., 2007 |
| WTCH_2 | Actively seek out information about health. |
| WTCH_3 | Quick to make an appointment to talk with physician when not well. |
| WTCH_4 | Experience difficulties communicating successfully with health care providers. |
| WTCH_5 | Competent communicator when talking about health issues. |
| **Willingness to communicate** | | |
| WTC_1 | Present a talk to a group of strangers. | McCroskey (1992) |
| WTC_2 | Talk in a small group of strangers. |
| WTC_3 | Talk with a stranger while standing in line. |
| WTC_4 | Talk in a large meeting of strangers. |
