# Supplementary material for: The Influence of Perceived Social Presence on the Willingness to Communicate in Mobile Medical Consultations: Experimental Study
Source: J Med Internet Res. 2022 May 11;24(5):e31797. doi: 10.2196/31797 (PMC9133978; doi:10.2196/31797)
Supplement: Multimedia Appendix 2 [file jmir_v24i5e31797_app2.docx]

**Multimedia Appendix 2. Questionnaire.**

*Thanks for your participation in the beta version of the self-examination of COVID infection. Now you’re invited to fill out the questionnaire. All the data are for research only and your personal details will not be released. Please answer the questions according to your personal feelings. Thanks for your support!*

1.Have you participated in any similar online self-examination of COVID infection before?

- Yes
- No

Previous Page 1/6 Next

*The following 5 questions are for your overall impression of the physician. There is no right or wrong answer. Please choose the description that matches your true feelings. 1 means strongly disagree, 3 means neutral, 5 means strongly agree.*

1. There is a sense of human contact when communicated with the online physician.

① ② ③ ④ ⑤

2. There is a sense of personalness from the online physician.

① ② ③ ④ ⑤

3. There is a sense of sociability when communicated with the online physician.

① ② ③ ④ ⑤

4. There is a sense of human warmth when communicated with the online physician.

① ② ③ ④ ⑤

5.There is a sense of human sensitivity when communicated with the online physician.

① ② ③ ④ ⑤

Previous Page 2/6 Next

*The following 4 questions are for your feelings during the self-examination process. There is no right or wrong answer. Please choose a description that matches your true feelings. 1 means strongly disagree, 3 means neutral, 5 means strongly agree.*

1. While participating in a conversation with a new online physician I am not nervous.

① ② ③ ④ ⑤

2. I was very tense and nervous when communicating with this online physician.

① ② ③ ④ ⑤

3. I am not afraid to speak up in medical interviews with this online physician.

① ② ③ ④ ⑤

4. Overall, I am very calm and relaxed when talking to this online physician.

① ② ③ ④ ⑤

Previous Page 3/6 Next

*The following 6 questions are about your communication with the physician during the examination. There is no right or wrong answer. Please choose a description that matches your true feelings. 1 means strongly disagree, 3 means neutral, 5 means strongly agree.*

1. I did a good job of presenting important history associated with my medical problem.

① ② ③ ④ ⑤

2. I did a good job of describing the symptoms of my medical problem.

① ② ③ ④ ⑤

3. I did a good job of answering the doctor’s questions thoroughly.

① ② ③ ④ ⑤

4. I did a good job of answering the doctor’s questions honestly.

① ② ③ ④ ⑤

5. I did a good job of contributing to a trusting relationship.

① ② ③ ④ ⑤

6. I did a good job of being open and honest.

① ② ③ ④ ⑤

Previous Page 4/6 Next

*The following 5 questions are about your feelings during your communication with the physician. There is no right or wrong answer. Please choose a description that matches your true feelings. 1 means strongly disagree, 3 means neutral, 5 means strongly agree.*

1. Comfortable talking about health with healthcare providers.

① ② ③ ④ ⑤

2. Actively seek out information about health.

① ② ③ ④ ⑤

3. Quick to make an appointment to talk with physician when not well.

① ② ③ ④ ⑤

4. Experience difficulties communicating successfully with health care providers.

① ② ③ ④ ⑤

5. Competent communicator when talking about health issues.

① ② ③ ④ ⑤

Previous Page 5/6 Next

*Please indicate the probability of the following four scenarios happening to you by a percentage, 0% is impossible, 100% is very likely*

1. Present a talk to a group of strangers：______%

2. Talk in a small group of strangers：______%

3. Talk with a stranger while standing in line：______%

4. Talk in a large meeting of strangers：______%

Previous Page 6/6 Next
